# Selective inhibition of autoimmune exacerbation while preserving the anti-tumor clinical benefit using IL-6 blockade in a patient with advanced melanoma and Crohn’s disease: a case report

**DOI:** 10.1186/s13045-016-0309-7

**Published:** 2016-09-05

**Authors:** Marc Uemura, Van A. Trinh, Cara Haymaker, Natalie Jackson, Dae Won Kim, James P. Allison, Padmanee Sharma, Luis Vence, Chantale Bernatchez, Patrick Hwu, Adi Diab

**Affiliations:** 1 DDepartment of Melanoma Medical Oncology, University of Texas-MD Anderson Cancer Center, 1515 Holcolmbe Blvd., Houston, TX 77030 USA; 2 Department of Immunology, University of Texas-MD Anderson Cancer Center, 1515 Holcombe Blvd., Houston, TX 77030 USA; 3 Department of Genitourinary Medical Oncology, University of Texas-MD Anderson Cancer Center, 1515 Holcombe Blvd., Houston, TX 77030 USA

**Keywords:** Checkpoint inhibitors, PD-1, CTLA-4, Pembrolizumab, Tocilizumab, Crohn’s disease, Metastatic melanoma

## Abstract

**Background:**

Novel immunotherapies, or checkpoint inhibitors, targeting programmed cell death protein-1 (PD-1) and cytotoxic T lymphocyte-associated antigen-4 (CTLA-4) have significantly improved outcomes for patients with numerous different cancer types. However, owing to their exclusion from clinical trials and risk for autoimmune exacerbation on these treatments, the impact on safety and degree of toxicity of these potentially life-prolonging therapies is not well characterized in patients with an underlying autoimmune disease or previous organ transplant.

**Case presentation:**

We report a case of a patient with advanced melanoma and refractory Crohn’s disease who was treated concurrently with *pembrolizumab* (anti-PD-1 antibody) and *tocilizumab* (anti-interluekin-6 receptor antibody). This novel treatment strategy was well tolerated and did not result in Crohn’s disease exacerbation for at least 16 weeks. Importantly, this treatment resulted in marked, durable antitumor responses.

**Conclusions:**

This outcome suggests that targeted immunosuppression combined with checkpoint inhibitors may hold promise as a treatment strategy for this unique patient population and may warrant additional study.

## Background

Checkpoint inhibitor (CPI) therapies targeting PD-1 and CTLA-4 have improved survival in patients with metastatic melanoma [1]. Immune-related adverse events (irAEs) are the most common toxicity associated with CPI therapies. IrAEs can affect any organ and result from immune dysregulation targeting normal tissue. Resultantly, patients with pre-existing autoimmunity are routinely excluded from CPI clinical trials for fear of exacerbating their underlying autoimmune condition and have limited treatment options. A notable, recently reported small retrospective review of 30 patients with pre-existing autoimmunity and advanced melanoma treated with *ipilimumab* (anti-CTLA-4 antibody) demonstrated that 27 % developed an autoimmune exacerbation and 33 % developed conventional grade 3–5 irAEs [2] including death. In specific relevance to this case, six of those patients had inflammatory bowel disease and two of them experienced a grade 3–5 irAE. Therefore, determining how to safely deliver immunotherapies to this unique population without exacerbating their autoimmune condition poses a significant clinical challenge and remains an unmet medical need.

Previous studies report Th-17, a helper T cell subset that releases interleukin-17 (IL-17), as a key mediator of many autoimmune diseases, including inflammatory bowel disease and CPI-induced colitis [3–5]. Importantly, IL-6 plays an essential role in inducing Th-17 from naïve CD4^+^ T cells [6]. Because of this, there has been recent interest in targeting this differentiated T cell pathway as novel therapy for autoimmunity [3]. In addition, IL-6 blockade has shown efficacy in reversing cytokine release syndrome, a clinical by-product of excessive immune activation seen with adoptive T cell therapies [7, 8], and has also shown preliminary efficacy against Crohn’s disease in an early pilot trial [9]. Here, we report a case in which *pembrolizumab* (anti-PD-1 antibody) was co-administered with *tocilizumab*, an anti-IL-6 receptor antibody that is FDA-approved for the treatment of rheumatoid arthritis, juvenile idiopathic arthritis, and polyarticular juvenile rheumatoid arthritis, in a patient with concomitant advanced melanoma and refractory Crohn’s disease. The patient showed a significant, durable antitumor response with limited Crohn’s disease exacerbation. This suggests that anti-PD-1 therapies, when combined with selective immune inhibitors, can have clinical benefit while possibly delaying autoimmune exacerbation in patients with concurrent advanced melanoma and Crohn’s disease.

## Case presentation

A 49-year-old woman with history of severe, refractory Crohn’s disease was diagnosed with stage IIA cutaneous left shoulder melanoma (Breslow thickness 2.8 mm without ulceration) in 2013. She previously had experienced multiple Crohn’s-related fistulas and required anti-TNF alpha therapy. She underwent wide local excision with sentinel lymph node biopsy showing no residual melanoma and was then placed on surveillance. In January 2015, she developed multiple extremity skin nodules and presented to our center. On presentation, her Crohn’s disease was managed with immunosuppressive therapy including 6-mercaptopurine and low-dose oral prednisone. Her Crohn’s disease was under moderate control and she experienced only mild diarrheal symptoms. Biopsies confirmed metastatic melanoma and mutational analyses revealed wild-type *BRAF*, *NRAS*, and *c-KIT*. Staging evaluation revealed multiple brain, liver, and lung metastases. She underwent stereotactic brain radiosurgery and initiated chemotherapy. Frontline anti-PD-1 or anti-CTLA-4 therapies were not chosen over concerns about exacerbating her Crohn’s disease (anti-PD-1 therapies are notably associated with Th-17 induction [10]). In May 2015, restaging imaging showed progressive brain and multi-organ metastases after receiving two cycles of chemotherapy.

**Fig. 1:**
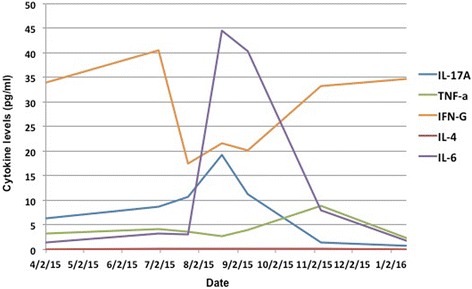
Correlative immune studies showing cytokine levels (measured in pg/ml) in peripheral blood at specific time points with combination therapy involving pembrolizumab and tocilizumab

She then initiated whole brain radiotherapy immediately followed by concurrent pembrolizumab (administered IV at 2 mg/kg every 21 days) and tocilizumab (administered IV at 8 mg/kg IV every 21 days) in July 2015 after discontinuing her previous immunosuppressive regimen. Anti-TNF agents and corticosteroids were avoided over concerns about possibly abrogating the anti-tumor response to anti-PD-1 agents, a phenomenon seen in a previous clinical trial that evaluated ipilimumab and corticosteroids in patients with metastatic melanoma to the brain [11]. After two treatment doses, significant anti-tumor responses were seen in the brain, liver, lung, and subcutaneous lesions without clinical evidence of Crohn’s disease exacerbation. Peripheral blood analysis demonstrated an expected increase in IL-6 but without a significant increase in IL-17, suggesting possible suppression of Th-17 induction (Fig. 1). Interestingly, tocilizumab did not inhibit the effector CD4^+^ or CD8^+^ T cells as demonstrated by immune phenotyping.

**Fig. 2:**
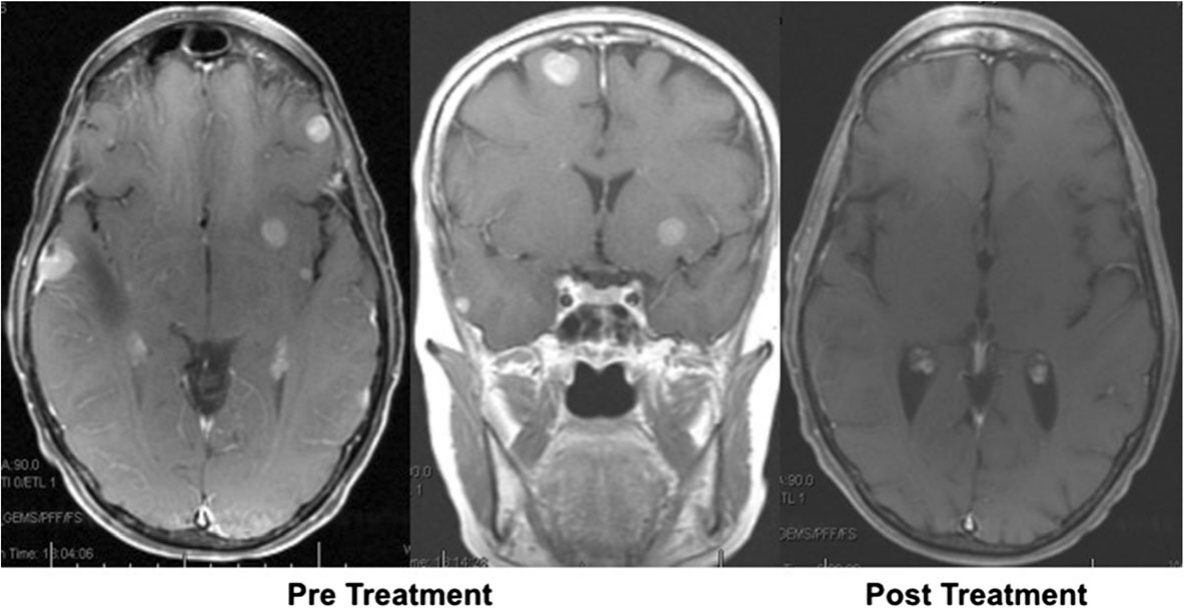
Pre- and post-treatment MRI findings showing near complete response to combination therapy with pembrolizumab and tocilizumab. Notably, the patient initially had 18 intracranial metastases that were completely resolved

Sixteen weeks after initiating therapy, she presented with an intra-abdominal abscess requiring drainage and antibiotics. We discontinued pembrolizumab and tocilizumab over concerns for possible Crohn’s disease exacerbation and initiated adalimumab, an anti-TNF monoclonal antibody. She subsequently recovered uneventfully. In January 2016, despite being off systemic therapy for 3 months, restaging evaluation showed near complete response of all metastatic sites, including the brain (Fig. 2). Pembrolizumab was then re-started along with concurrent adalimumab and her most recent imaging in March 2016 showed complete response to therapy.

### Discussion

As a tertiary cancer center, we routinely receive clinically challenging referrals—the patient described in this case report is a salient example of this. Our case illustrates that the strategy of co-administering pembrolizumab with an agent specifically targeting the IL-6 - Th-17 - IL-17 pathway, in this case, tocilizumab, in a patient with advanced melanoma and Crohn’s disease may result in significant anti-tumor effects while possibly preventing/delaying autoimmune consequences. Additionally, for our patient, this combination was well tolerated for at least 16 weeks with limited signs or symptoms of Crohn’s exacerbation (i.e., diarrhea, fevers, and abdominal pain).

The patient’s peripheral blood analysis demonstrated that combination therapy resulted in an expected increase in IL-6 levels. This finding was previously reported in a study of adult patients with rheumatoid arthritis or Castleman’s disease who received tocilizumab and is thought to result from decreased IL-6R consumption of IL-6 [12]. However, what is more intriguing is that there was a little change in IL-17 levels, which again suggests that there was a limitation of Th-17 induction as they are the major producers of IL-17 (Fig. 1). Considering their documented role in both irAEs and Crohn’s disease pathogenesis [5], blocking Th-17 induction with tocilizumab could be one possible explanation as to why our patient demonstrated delayed exacerbation of her underlying Crohn’s disease. Of course, additional studies are needed to confirm this finding.

Furthermore, targeting specific aspects of the immune response with this strategy has other significant consequences. It is important to note, for example, that Th-17 cells have been shown to be resistant to glucocorticoids, which may explain why some patients who develop irAEs or autoimmune exacerbation do not respond to corticosteroids [13]. Therefore, a strategy which specifically focuses on preventing Th-17 cell induction may diminish the need for broad immune suppression with corticosteroids. As mentioned, using corticosteroids to treat immune toxicities may also abrogate the anti-tumor effects of CPI therapies, making their use possibly detrimental for patients who develop these side effects [11].

Recently, numerous studies have focused on targeting this immune pathway in the treatment of autoimmune diseases. Interleukin-17A inhibitors have been studied in large clinical trials in plaque psoriasis and ankylosing spondylitis. In fact, *secukinumab* and *ixekizumab*, both IL-17A antibodies, have been FDA-approved for the treatment of plaque psoriasis based on phase 3 trials [14, 15]. Whether or not these agents have a role in treating patients with autoimmunity and concurrent cancer has yet to be determined.

## Conclusions

This case illustrates that co-administration of anti-PD-1 with anti-IL-6R in patients with advanced melanoma and Crohn’s disease can be well tolerated and may attenuate or delay autoimmune exacerbation without impacting a positive anti-tumor effects. Because this is a report of one patient only, the results described are purely descriptive and we are unable to draw any definitive conclusions about the impact of this intervention on disease-specific outcomes. In summary, the IL-6 - Th-17 - IL-17 pathway may play a pathogenic role in mediating the irAEs and/or autoimmune exacerbations of patients with an underlying autoimmune disease treated with immunotherapy and deserves further study.

## Abbreviations

IL: Interleukin
irAE: Immune-related adverse events
CPI: Checkpoint inhibitor
PD-1: Programmed cell death protein-1
CTLA-4: Cytotoxic T lymphocyte-associated antigen-4
